# Evaluation of visual function within the central 10 degrees using IMOvifa™ 24plus (1-2)

**DOI:** 10.1371/journal.pone.0323630

**Published:** 2025-05-12

**Authors:** Yuki Takagi, Ryo Asano, Kana Yamashita, Yukihiro Sakai, Sho Yokoyama, Kei Ichikawa, Kazuo Ichikawa

**Affiliations:** 1 Chukyo Hospital 1-10, Nagoya, Aichi, Japan; 2 Chukyo Eye Clinic 12-22, Nagoya, Aichi, Japan; 3 Asano Eye Clinic, Nagoya, Aichi, Japan; Seirei Hamamatsu General Hospital, JAPAN

## Abstract

**Purpose:**

The IMOvifa™ perimeter with a 24plus (1-2) testing mode has additional measurement points within the central 10 degrees, which may help evaluate the visual field within this area. Here, we comparatively evaluated the IMOvifa™ 24plus (1-2) and HFA 10-2 for the first time.

**Methods:**

We included 30 patients (48 eyes) who underwent HFA 24-2 Swedish Interactive Threshold Algorithm Standard and IMOvifa™ 24plus (1-2) Ambient Interactive Zippy Estimated tests on the same day and HFA 10-2 within six months. We used Spearman’s rank correlation coefficient to analyze the mean deviation (MD) and pattern standard deviation (PSD) between HFA 10-2 and IMOvifa™. The central 10-degree visual field was divided into four sectors, and concordance of visual field defects between IMOvifa™ 24plus (1-2) and HFA 10-2 was evaluated using kappa analysis. Additionally, all sectors showing a sensitivity of 0 dB on the HFA 24-2 were assessed for the presence and agreement of residual visual field in HFA 10-2 and IMOvifa™ 24plus (1-2).

**Results:**

The MD (0.843/0.804) and PSD (0.852/0.763) of IMOvifa™ 24plus (1-2) and HFA 24-2 correlated strongly with those of HFA 10-2. Regarding the ability to detect visual field defects within the central 10 degrees, agreement with HFA 10-2 was κ = 0.715 (0.611,0.819) and 0.754 (0.654,0.854) for IMOvifa™ 24plus (1-2) and HFA 24-2, respectively. In the evaluation of residual visual field, IMOvifa™ 24plus (1-2) detected residual visual function in 100% of cases where HFA 10-2 indicated residual function.

**Conclusion:**

The IMOvifa™ 24plus (1-2) may have a higher ability to detect defects in certain areas of the visual field, compared with HFA 24-2, and may also detect residual visual function. However, the IMOvifa™ 24plus (1-2) is difficult to substitute for the 10-2 test, as the 10-2 test is necessary for evaluating visual field defects within the central 10 degrees.

## Introduction

The IMO and IMOvifa™ (CREWT Medical Systems, Tokyo, Japan) are advanced static perimetry devices designed to perform visual field testing with both eyes open. Studies have found a strong correlation between results obtained using these devices and those using the Humphrey Field Analyzer (HFA) (Carl Zeiss Meditec, Dublin, CA, USA). Moreover, IMOvifa™ reportedly completes testing in a shorter duration than the HFA [1–4]. A notable feature of IMOvifa™ is its capability for binocular random single-eye testing, wherein visual stimuli are presented randomly to each eye while both eyes remain open. This approach enables simultaneous parallel testing of both eyes and has been shown to yield highly reproducible results that are comparable to monocular testing conducted using the HFA [5]. Furthermore, in binocular random single-eye testing, patients are unable to discern which eye is being tested, a characteristic that may prove valuable for diagnosing psychogenic visual field defects and malingering [6,7].

The central 10 degrees of the visual field are critical for visual function, as they directly correlate with visual acuity. However, in the widely used 30-2 or 24-2 test conditions, only 12 measurement points fall within this region, which limits the ability to comprehensively assess visual function. Studies that compared the visual field test results of HFA 24-2 and HFA 10-2 have indicated that HFA 24-2 fails to detect 16–52.5% of visual field defects identified by HFA 10-2 [8,9] and is less effective in monitoring the progression of visual field defects [10]. To address these limitations, various strategies have been proposed to improve the detection of abnormalities within the central 10 degrees [11–13].

**Fig 1:**
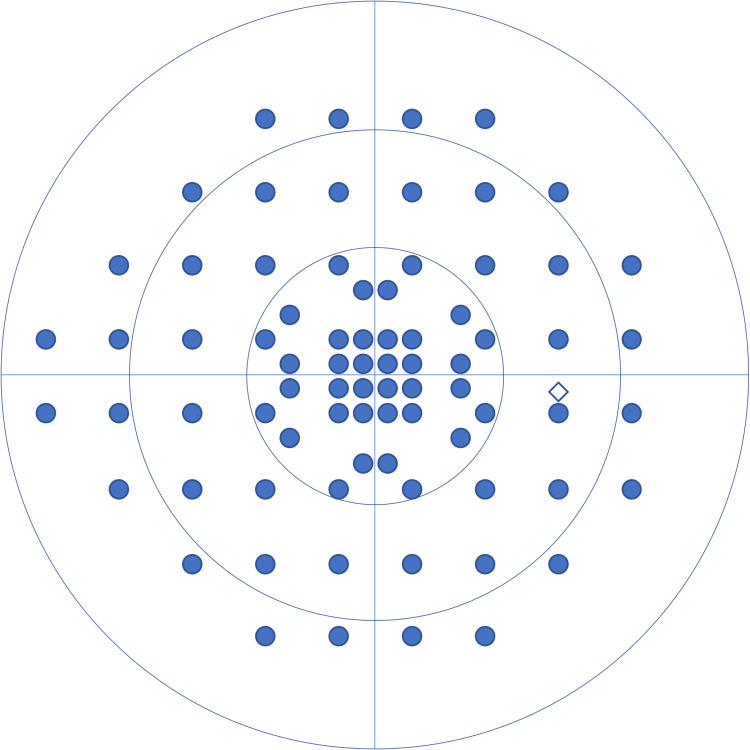
Arrangement of measurement point in IMOvifa™ 24plus (1-2). The 24plus (1-2) adds 24 measurement points symmetrically within the central 10 degrees to the 24-2 grid.

In response, the HFA introduced the 24-2c test mode to enhance the detection of visual field defects within the central 10 degrees. Studies have shown that, compared with 24-2, the 24-2c test mode significantly improves the correlation between macular structure and function [14,15] and is therefore more effective in detecting visual field defects [15–19]. Similarly, the IMO and IMOvifa™ devices feature a test mode called 24plus (1-2), which incorporates additional measurement points within the central 10 degrees (Fig 1). A study that compared IMO 24plus (1-2) and HFA 30-2 indicated a high correlation in results, such as mean deviation (MD) values [3]. However, to the best of our knowledge, no study has compared IMOvifa™ 24plus (1-2) with HFA 10-2 in evaluating visual function within the central 10 degrees.

Therefore, in this study, we aimed to compare the results of IMOvifa™ 24plus (1-2) with those of HFA 10-2 and to assess visual function within the central 10 degrees.

## Materials and methods

### Patient and study design

This prospective study was conducted at the glaucoma outpatient department of Chukyo Eye Clinic between June 2023 and March 2024, involving participants who provided written informed consent. The study was approved by the Ethics Committee of Chukyo Eye Clinic (approval number: 20230530052) and conducted in accordance with the Declaration of Helsinki. Written informed consent was obtained from all participants following a detailed explanation provided in writing.

Eligible participants were aged ≥20 years, had a history of multiple visual field tests, and were diagnosed with glaucoma or pre-perimetric glaucoma. The diagnosis of glaucoma or pre-perimetric glaucoma was made based on the following criteria: glaucomatous optic nerve changes observed on fundus photography and optical coherence tomography (Cirrus, Carl Zeiss Meditec, Dublin, CA, USA), including a horizontal cup-to-disc (C/D) ratio of ≥ 0.7, rim notching, rim width of ≤ 0.1, and retinal nerve fiber layer defects. Cases were classified as glaucoma if corresponding glaucomatous visual field defects were identified using the 24-2 SITA Standard protocol on the HFA. Cases without visual field defects were classified as pre-perimetric glaucoma.

Exclusion criteria included any comorbid conditions that could cause visual field abnormalities, such as optic neuritis, retinal disease, intracranial disease, or psychogenic disorders. No restrictions were applied regarding disease stage, glaucoma type, refractive error, or corrected visual acuity.

### Visual field tests

Participants who provided informed consent underwent visual field testing on the same day using the HFA 24-2 SITA Standard and the IMOvifa™ 24plus (1-2) Ambient Interactive Zippy Estimated (AIZE). The HFA 10-2 SITA Standard was subsequently performed within six months. Participants were excluded if any of the visual field tests exhibited a fixation loss rate >20%, false positive rate >20%, or false negative rate >33%. During the HFA tests, the non-tested eye was occluded to ensure accurate monocular measurements. In contrast, the IMOvifa™ 24plus (1-2) test employed binocular random single-eye testing, which allowed for both eyes to remain open throughout the examination without occlusion.

### Statistical analysis

We evaluated the MD and pattern standard deviation (PSD) values obtained with HFA 10-2, IMOvifa™ 24plus (1-2), and HFA 24-2 using Spearman’s rank correlation coefficient to determine the degree of correlation. Additionally, the reliability of MD and PSD measurements of HFA 10-2 versus those of HFA 24-2 and IMOvifa™ was assessed using intra-class correlation coefficient (ICC).

**Fig 2:**
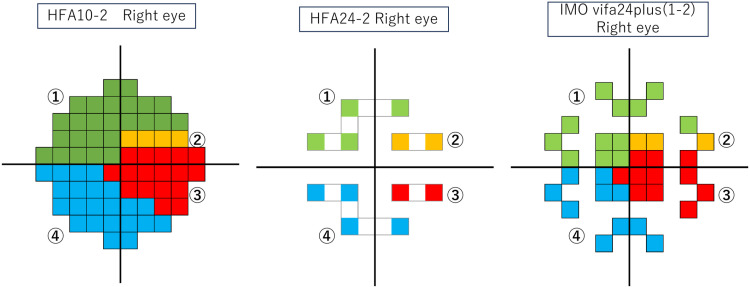
Visual field divided within the central 10 degrees into four sectors based on the Nakanishi Map for HFA 10-2, IMOvifa™ 24plus (1-2), and HFA 24-2. The visual fields divide so that Sector 1 is superior parafoveal scotoma area, Sector 2 is superior cecocentral scotoma area, Sector 3 is overlapping superior and inferior hemifields, including the cecocentral scotoma area, Sector 4 is inferior parafoveal scotoma area.

To assess the ability to detect visual field defects within the central 10 degrees, we divided this region into four sectors based on the Nakanishi Map [20] for HFA 10-2, IMOvifa™ 24plus (1-2), and HFA 24-2 (Fig 2). Using pattern deviation (PD) values, we performed kappa analysis to evaluate the concordance of visual field defects across the entire visual field and within each sector between HFA 10-2 and both HFA 24-2 and IMOvifa™ 24plus (1-2). We defined visual field abnormalities as follows: for HFA 10-2, the presence of three contiguous abnormal points with P < 1%, 5%, 5% or P < 2%, 2%, 5% within the sectors on the PD plot; for IMOvifa™ 24plus (1-2) and HFA 24-2, the presence of at least one abnormal point with P < 1% within the sectors on the PD plot. Since some sectors in HFA 24-2 and IMOvifa™ 24plus (1-2) contain at most two adjacent test points, we defined visual field abnormalities as the presence of at least one point with P < 1% in those sectors.

To evaluate the ability to detect residual visual function within the central 10 degrees, we identified cases wherein all measurement points in a given sector exhibited a sensitivity of 0 dB on the HFA 24-2. Among these cases, we extracted those with residual visual fields detected in the corresponding sectors of the HFA 10-2. We then evaluated the proportion of these cases in which residual visual fields were also identified in the corresponding sectors of the IMOvifa™ 24plus (1-2).

Additionally, we performed a power analysis for the primary outcome measures, including MD and PSD, using Spearman’s rank correlation coefficient.

All statistical analyses were performed using SPSS (version 29.0; IBM Corp., Armonk, NY., USA), and statistical significance was set at P < 0.05.

## Results

We included 30 patients (14 men and 16 women) with 48 eyes (21 eyes in men, 27 in women; 24 right eyes and 24 left eyes). The clinical characteristics of the participants are shown in Table 1. The mean age was 63.1 ± 10.7 years, with a mean refractive error of −3.45 ± 4.12 diopters and a corrected visual acuity of −0.063 ± 0.18 logMAR. The cohort included 37 phakic eyes and 11 eyes with intraocular lenses (IOL). Regarding glaucoma subtypes, 45 eyes had primary open-angle glaucoma (POAG), two had pseudoexfoliation glaucoma (PEG), and one had secondary open-angle glaucoma (SOAG).

**Table 1:** Clinical characteristics of the participants.

Characteristics	Total (n = 48)	Spearman’s rank correlation test with HFA 10-2 MD (P)	Spearman’s rank correlation test with HFA 10-2 PSD (P)
Age, years	63.1 ± 10.7	-0.277 (0.056)	0.401 (0.005)
Sex (Female, eyes (%))	28 (53.33)	–	–
Side (R, eyes (%))	24 (50)		
Spherical equivalent (D)	−3.45 ± 4.12	−0.122 (0.411)	0.203 (0.167)
BCVA (logMAR)	−0.063 ± 0.18	−0.348 (0.015)	0.262 (0.072)
Lens (Phakia, number (%))	37 (77.08)	–	–
Glaucoma type	POAG:45, PEG2, SOAG1		
HFA24-2 VFI(%)	83.88 ± 19.40	0.811 (<0.001)	−0.865 (<0.001)
HFA24-2 MD (dB)	−5.54 ± 6.59	0.801 (<0.001)	−0.814 (<0.001)
HFA24-2 PSD (dB)	6.75 ± 4.81	−0.634 (<0.001)	0.764 (<0.001)
IMOvifa™ 24plus (1-2) VFI (%)	84.77 ± 18.59	0.822 (<0.001)	−0.878 (<0.001)
IMOvifa™ 24plus (1-2) MD (dB)	−5.62 ± 6.53	0.835 (<0.001)	−0.865 (<0.001)
IMOvifa™ 24plus (1-2) PSD (dB)	7.08 ± 4.79	−0.714 (<0.001)	0.840 (<0.001)
HFA10-2 MD (dB)	−4.88 ± 6.67	–	–
HFA10-2 PSD (dB)	5.29 ± 4.81	–	–

diopter (D), Best corrected visual acuity (BCVA), visual field index (VFI), mean deviation (MD), pattern standard deviation (PSD), primary open angle glaucoma (POAG), pseudo-exfoliation glaucoma (PEG), secondary glaucoma (SG)

The MD and PSD values obtained from the HFA 10-2, IMOvifa™ 24plus (1-2), and HFA 24-2 tests were as follows: MD (−4.88 ± 6.67, −5.62 ± 6.53, −5.54 ± 6.59) and PSD (5.29 ± 4.81, 7.08 ± 4.79, 6.75 ± 4.81). Spearman’s rank correlation coefficients for MD and PSD between HFA 10-2 and IMOvifa™ 24plus (1-2)/HFA 24-2 were as follows: MD: 0.835/0.801 and PSD: 0.840/0.764, with all correlations being statistically significant (all P < 0.001) (Table 1). The ICC for MD of HFA 10-2 versus MD of HFA 24-2 and MD of IMOvifa™ was 0.862 and 0.898, with 95% confidence intervals (CIs) of (0.767–0.920) and (0.824–0.924), respectively (P < 0.001), demonstrating high reliability. Similarly, the ICC for PSD of HFA 10-2 versus PSD of HFA 24-2 and PSD of IMOvifa™ was 0.712 and 0.804, with 95% CIs of (0.509–0.834) and (0.499–0.910), respectively (P < 0.001), indicating high reliability.

In the power analysis, the detection rates for HFA 10-2 MD and PSD were 46.5% and 78.3% for age, 13.0% and 27.7% for refraction, and 65.8% and 42.5% for BCVA, respectively. The detection rates were 100% for HFA 24-2 visual field index (VFI), HFA 24-2 MD, IMOvifa™ 24plus (1-2) VFI, IMOvifa™ 24plus (1-2) MD, and IMOvifa™ 24plus (1-2) PSD. For HFA 24-2 PSD, the detection rates were 99.6% and 100%.

Table 2 shows the detection ability for visual field defects within the central 10 degrees. The results for HFA 10-2 and IMOvifa™ 24plus (1-2) were sensitivity of 78.57%, specificity of 91.8%, κ value of 0.715 (0.611,0.819), and AUC of 0.848 (0.782, 0.914). For HFA 10-2 and HFA 24-2, sensitivity was 75.76%, specificity 96.61%, κ value was 0.754 (0.654,0.854), and AUC 0.862 (0.797, 0.927).

**Table 2:** Evaluation of the ability to detect visual field defects within the central 10 degrees in HFA 24-2 and IMOvifa™ 24Plus (1-2).

	Sensitivity (%)	Specificity (%)	κ analysis (95% confidence interval)	AUC (95% confidence interval)
Between HFA10-2 and IMOvifa™24plus(1-2)				
entire visual field within the central 10 degrees	78.57	91.80	0.715 (0.611,0.819)	0.848 (0.782,0.914)
Sector 1	88.00	91.30	0.792 (0.620,0.964)	0.891 (0.786,0.996)
Sector 2	70.0	92.11	0.621 (0.345,0.897)	0.822 (0.644,1.0)
Sector 3	77.78	89.74	0.622 (0.348,0.896)	0.835 (0.664,1.0)
Sector 4	73.08	95.45	0.671 (0.469,0.873)	0.831 (0.707,0.956)
Between HFA10-2 and HFA24-2				
entire visual field within the central 10 degrees	75.76	96.61	0.754 (0.654,0.854)	0.862 (0.797,0.927)
Sector 1	86.96	95.65	0.826 (0.663,0.989)	0.913 (0.818,1.0)
Sector 2	90.0	97.22	0.872 (0.700,1.0)	0.936 (0.822,1.0)
Sector 3	55.56	97.30	0.605 (0.295,0.915)	0.764 (0.553,0.975)
Sector 4	66.67	95.45	0.613 (0.397,0.827)	0.811 (0.680,0.941)

Table 2 shows the results for each sector. Kappa analysis for HFA 10-2 and both IMOvifa™ 24plus (1-2) and HFA 24-2 by sector were as follows: Sector 1: 0.792 (0.620,0.964), 0.826 (0.663,0.989); Sector 2: 0.621 (0.345,0.897), 0.872 (0.700,1.0); Sector 3: 0.622 (0.348,0.896), 0.605 (0.295,0.915); and Sector 4: 0.671 (0.469,0.873), 0.613 (0.397,0.827).

To evaluate residual visual function within the central 10 degrees, four eyes (six sectors) in which all measurement points within each sector exhibited a sensitivity of 0 dB on HFA 24-2 were selected. Residual visual function was detected in all six sectors using HFA 10-2, and IMOvifa™ 24plus (1-2) also successfully identified residual visual fields in these sectors.

**Fig 3:**
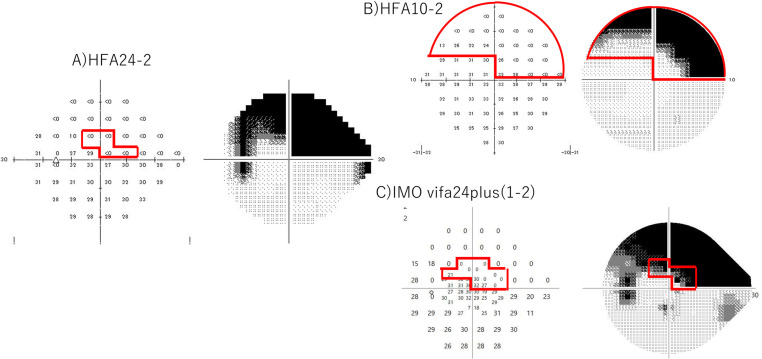
Example of a case with residual visual function within the central 10 degrees. (A) shows that HFA 24-2 showed a sensitivity of 0 dB for all measurement points in Sector 1. (B) shows that HFA 10-2 detected residual visual fields in the more central area of Sector 1. (C) shows that IMOvifa™ 24plus (1-2) similarly showed residual visual function.

A representative case demonstrating residual visual function within the central 10 degrees is presented in Fig 3. In this case, HFA 24-2 indicated a sensitivity of 0 dB across all measurement points in Sector 1. However, HFA 10-2 detected residual visual fields in the more central area of Sector 1, and IMOvifa™ 24plus (1-2) similarly identified residual visual function. Notably, in IMOvifa™ 24plus (1-2), the points corresponding to the conventional 24-2 measurement showed 0 dB, while residual visual fields were detected at the additional measurement points introduced by the 24plus (1-2) mode.

## Discussion

For the MD and PSD of HFA 10-2, the VFI, MD, and PSD of both HFA 24-2 and IMOvifa™ 24plus (1-2) correlated strongly and significantly. The correlation tended to be slightly stronger with IMOvifa™ 24plus (1-2). IMOvifa™ 24plus (1-2) includes 36 measurement points within the central 10 degrees, compared with the 12 points in HFA 24-2. This expanded measurement area allows parameters such as MD to more effectively reflect the presence or absence of visual field defects within the central 10 degrees. These findings suggest that IMOvifa™ 24plus (1-2) may provide better estimations of HFA 10-2 results than standard HFA 24-2.

Regarding the detection of visual field defects within the central 10 degrees, both IMOvifa™ 24plus (1-2) and HFA 24-2 showed high agreement across the entire visual field. However, sensitivity, specificity, and kappa analysis results slightly favored HFA 24-2 over IMOvifa™ 24plus (1-2). A study that examined the sensitivity of HFA 24-2 in detecting abnormalities observed on HFA 10-2 indicated sensitivities of 66% for MD < 5%, 83% for PSD, 76% for the glaucoma hemifield test (GHT), 88% for cluster criteria (three neighboring points at 5%, 5%, and 1% or 5%, 2%, and 2% probability or worse within a hemifield on TD or PD plots, with only one point allowed on the edge of the 24-2), and 85% for macular points (MPs) (within ±10°: one point at 1% or two at 2% within a hemifield on TD or PD) [8]. The definition of visual field abnormality in this study is similar to that of MPs in previous reports; however, the sensitivity is slightly lower. This discrepancy may stem from the fact that MPs, even two contiguous points at 2%, are considered to indicate a visual field abnormality, and that while earlier studies divided the visual field into the upper and lower hemifields, the present study subdivided the visual field into sectors, applying stricter criteria.

Kappa analysis in a previous study [17] indicated moderate agreement, which is lower than the kappa observed in this study for HFA 24-2. This discrepancy likely arises from differences in the definition of visual field defects. In that report [17], visual field defects were defined as having three or more contiguous points with reduced sensitivity, making the criteria stricter than those in this study. Therefore, the definition of visual field abnormalities should be standardized, and the findings should be re-evaluated in future studies.

Sector-by-sector analysis showed better agreement with HFA24-2 than with IMOvifa™24plus (1-2). In Sector 1, HFA24-2 performed slightly better than IMOvifa™ 24plus (1-2). Sector 2 had high agreement for HFA 24-2 but moderate agreement for IMOvifa™ 24plus (1-2). Both Sectors 3 and 4 were in moderate agreement; however, IMOvifa™ 24plus (1-2) was slightly better. Sensitivity and specificity patterns mirrored kappa, except for the specificity of Sector 3. These findings suggest that HFA 24-2 was better than IMOvifa™ 24plus (1-2), especially in Sector 2, which may have influenced the better agreement rate in the entire field of view for HFA 24-2. However, since IMOvifa™ 24plus (1-2) had more measurement points within the central 10° than HFA 24-2 (36 vs 12 as within 10°, 12 vs 4 for sector 1, 3 vs 2 for sector 2, 11 vs 2 for sector 3, and 10 vs 4 for sector 4), we assumed that kappa and other values would be better. The results for Sectors 3 and 4 were in line with this assumption, but the results for Sectors 1 and 2 were different.

One notable factor influencing IMOvifa™ results may be its binocular random single-eye testing method, which differs from the monocular testing approach of HFA. Several studies have examined the impact of this open-eye testing on visual sensitivity [21–24]. One study that used IMO in healthy individuals to compare sensitivity with and without monocular occlusion found that sensitivity was higher without occlusion [23]. Kumagai et al. classified eyes as “Better eye” or “Worse eye” based on visual acuity and foveal threshold, then compared visual sensitivity at the fovea and the four central points between monocular testing and binocular random single-eye testing using IMO [22]. Their findings indicated that visual sensitivity was higher in the binocular random single-eye test than in the monocular test for the Better eye, but lower for the Worse eye. This suggests that testing without monocular occlusion and with both eyes simultaneously, as well as the functional status of the non-tested eye can affect visual sensitivity, potentially leading to differences in results compared to HFA 24-2 or HFA 10-2. Therefore, these effects presumably caused the binocular random single-eye test of IMOvifa™ to fail to detect visual field abnormalities that would be detected in a single-eye-only visual field test with one eye occluded, resulting in lower sensitivity and kappa than HFA 24-2. However, IMOvifa™ 24plus (1-2) had better sensitivity and kappa, even though similar effects were presumably present in Sectors 3 and 4. The sensitivity and kappa of IMOvifa™ 24plus (1-2) in Sector 2 were significantly lower than those of HFA 24-2. Therefore, the effects of IMO and IMOvifa™ on binocular random single-eye test sensitivity may vary depending on the location of the visual field, and more detailed studies using Pointwise and other methods are needed in the future.

HFA 24-2C is another test condition that, like IMOvifa™ 24plus (1-2), incorporates additional test points within the central 10 degrees. Several studies have examined the effectiveness of HFA 24-2C to detect abnormalities in this region [15–19]. Behera et al. compared the ability of HFA 24-2C and HFA 24-2 to detect abnormal points within the central 10 degrees and reported that HFA 24-2C detected an average of 5.5 more points on the TD plot and 2 more points on the PD plot [18]. Additionally, Nishizima et al. analyzed the abnormality detection performance of HFA 24-2C and HFA 24-2 by dividing the central 10-degree visual field into upper and lower halves and evaluating the results using receiver operating characteristic curves. They compared 22 test points in the central 10 degrees of HFA 24-2C with 4 and 12 points from HFA 24-2, and their findings indicated that HFA 24-2C demonstrated superior abnormality detection ability in both the upper and lower visual fields [19]. However, in the present study using IMOvifa™ 24plus (1-2), results suggested that its ability to detect visual field abnormalities within the central 10 degrees may be partially inferior to that of HFA 24-2. One possible explanation for this could be the influence of the binocular open-view testing condition, as mentioned earlier. Nevertheless, differences in the definition of visual field abnormalities and the analytical methods used between previous studies [18,19] and the present study prevent a direct comparison between IMOvifa™ 24plus (1-2) and HFA 24-2C. Therefore, further investigations under standardized conditions are needed to enable a direct comparison between IMOvifa™ 24plus (1-2) and HFA 24-2C.

Regarding the detection of residual visual function within the central 10 degrees, IMOvifa™ 24plus (1-2) detected residual visual fields in all sectors where HFA 10-2 identified residual function. This is likely due to the fact that IMOvifa™ considers additional measurement points within the central 10 degrees which may make it more useful than HFA 24-2 for evaluating residual visual fields within this area.

Several studies have examined the correlation between visual field function and structure; however, no unified conclusions have been reached [20,25–27]. In this study, we evaluated the central 10 degrees of the visual field using the Nakanishi Map [20], which divides the central 10 degrees into four sectors: VF1 (superior parafoveal scotoma area, Sector 1 in this study), VF2 (inferior parafoveal scotoma area, Sector 4), VF4 (overlapping superior and inferior hemifields, including the cecocentral scotoma area, Sector 3), and VF5 (superior cecocentral scotoma area, Sector 2). The Nakanishi Map divides the visual field and cpRNFL using enhanced en face analysis, evaluating corresponding sectors based on their correlation, which showed a moderate to high correlation. However, sectors surrounding the cpRNFL that corresponded to the visual field sectors also showed moderate correlations, which indicates that the structural segmentation may not be entirely distinct. Consequently, in some cases, structurally different areas may fall within the same sector, which could have influenced the findings in this study.

The limitations of this study include a relatively small sample size of 48 eyes, indicating the need for further studies with a larger number of cases. Additionally, no study thus far has directly compared HFA 24-2c and IMOvifa™ 24plus (1-2), highlighting the necessity of conducting a comparative study under consistent conditions. Furthermore, although binocular open-field testing with IMO is reportedly influenced by the “Better Eye” and “Worse Eye,” more detailed research is needed to clarify which areas of the visual field are most affected. In some cases, there was a six-month interval between the administration of HFA 24-2, IMOvifa™ 24plus (1-2), and HFA 10-2, raising the possibility that the progression of visual field defects within the central 10 degrees may have affected the results. Moreover, in this study, the definition of visual field abnormalities for HFA 10-2 differed from those for the other two tests, HFA 24-2 and IMOvifa™ 24plus (1-2). Since some sectors in HFA 24-2 and IMOvifa™ 24plus (1-2) contain at most two adjacent test points, we defined visual field abnormalities as the presence of at least one point with P < 1% in those sectors. As this may have influenced the results, future studies using a unified definition of visual field abnormalities are warranted. Finally, since the exclusion criteria in this study were not strictly defined, there are limitations in accuracy. Therefore, future studies should apply more stringent criteria to improve the accuracy of the research.

In conclusion, the current study showed that while IMOvifa™ 24plus (1-2) has limitations in detecting visual field defects within the central 10 degrees, it may offer higher detection capability than HFA 24-2 in certain areas of the visual field. It may also be useful for detecting residual visual function. However, substituting it for the HFA 10-2 test may be challenging, and performing the 10-2 test remains essential in cases where visual field defects within the central 10 degrees are suspected.

## References

[1] Matsumoto C, Yamao S, Nomoto H, Takada S, Okuyama S, Kimura S, et al. Visual field testing with head-mounted perimeter “imo”. PLoS One. 2016;11(8):e0161974. doi: 10.1371/journal.pone.0161974 27564382 PMC5001626

[2] Nakai Y, Bessho K, Shono Y, Taoka K, Nakai Y. Comparison of imo and Humphrey Field Analyzer perimeters in glaucomatous eyes. Int J Ophthalmol. 2021;14(12):1882–7. doi: 10.18240/ijo.2021.12.11 34926203 PMC8640777

[3] Kimura T, Matsumoto C, Nomoto H. Comparison of head-mounted perimeter (imo®) and Humphrey Field Analyzer. Clin Ophthalmol. 2019;13:501–13. doi: 10.2147/OPTH.S190995 30936681 PMC6422415

[4] Nishida T, Eslani M, Weinreb RN, Arias J, Vasile C, Mohammadzadeh V, et al. Perimetric comparison between the IMOvifa and Humphrey Field Analyzer. J Glaucoma. 2023;32(2):85–92. doi: 10.1097/IJG.0000000000002134 36223309

[5] Toyokuni H, Sakamoto M, Ueda K, Kurimoto T, Yamada-Nakanishi Y, Nakamura M. Test-retest repeatability of the imo binocular random single-eye test and Humphrey monocular test in patients with glaucoma. Jpn J Ophthalmol. 2023;67(5):578–89. doi: 10.1007/s10384-023-01007-5 37392238

[6] Goseki T, Ishikawa H, Shoji N. Bilateral concurrent eye examination with a head-mounted perimeter for diagnosing functional visual loss. Neuroophthalmology. 2016;40(6):281–5. doi: 10.1080/01658107.2016.1220593 27928419 PMC5120740

[7] Takagi Y, Yokoyama S, Yokoyama Y, Hozumi K, Kaga T. A case of functional visual loss diagnosed through bilateral randomized visual field testing with a trick method. Am J Ophthalmol Case Rep. 2023;32:101877. doi: 10.1016/j.ajoc.2023.101877 38161514 PMC10757184

[8] Grillo LM, Wang DL, Ramachandran R, Ehrlich AC, De Moraes CG, Ritch R, et al. The 24-2 visual field test misses central macular damage confirmed by the 10-2 visual field test and optical coherence tomography. Transl Vis Sci Technol. 2016;5(2):15. doi: 10.1167/tvst.5.2.15 27134774 PMC4849532

[9] Traynis I, De Moraes CG, Raza AS, Liebmann JM, Ritch R, Hood DC. Prevalence and nature of early glaucomatous defects in the central 10° of the visual field. JAMA Ophthalmol. 2014;132(3):291–7. doi: 10.1001/jamaophthalmol.2013.7656 24407153 PMC4204644

[10] Park SC, Kung Y, Su D, Simonson JL, Furlanetto RL, Liebmann JM, et al. Parafoveal scotoma progression in glaucoma: humphrey 10-2 versus 24-2 visual field analysis. Ophthalmology. 2013;120(8):1546–50. doi: 10.1016/j.ophtha.2013.01.045 23697959

[11] Wu Z, Medeiros FA, Weinreb RN, Zangwill LM. Performance of the 10-2 and 24-2 visual field tests for detecting central visual field abnormalities in glaucoma. Am J Ophthalmol. 2018;196:10–7. doi: 10.1016/j.ajo.2018.08.010 30099037 PMC6258276

[12] Chen S, McKendrick AM, Turpin A. Choosing two points to add to the 24-2 pattern to better describe macular visual field damage due to glaucoma. Br J Ophthalmol. 2015;99(9):1236–9. doi: 10.1136/bjophthalmol-2014-306431 25802251

[13] Ehrlich AC, Raza AS, Ritch R, Hood DC. Modifying the conventional visual field test pattern to improve the detection of early glaucomatous defects in the central 10°. Transl Vis Sci Technol. 2014;3(6):6. doi: 10.1167/tvst.3.6.6 25653884 PMC4315509

[14] Hong JW, Baek MS, Lee JY, Song MK, Shin JW, Kook MS. Comparison of the 24-2 and 24-2C visual field grids in determining the macular structure-function relationship in glaucoma. J Glaucoma. 2021;30(10):887–94. doi: 10.1097/IJG.0000000000001928 34387259

[15] Phu J, Kalloniatis M. Ability of 24-2C and 24-2 grids to identify central visual field defects and structure-function concordance in glaucoma and suspects. Am J Ophthalmol. 2020;219:317–31. doi: 10.1016/j.ajo.2020.06.024 32621896

[16] Phu J, Kalloniatis M. Comparison of 10-2 and 24-2C test grids for identifying central visual field defects in glaucoma and suspect patients. Ophthalmology. 2021;128(10):1405–16. doi: 10.1016/j.ophtha.2021.03.014 33722636

[17] Chakravarti T, Moghadam M, Proudfoot JA, Weinreb RN, Bowd C, Zangwill LM. Agreement between 10-2 and 24-2C visual field test protocols for detecting glaucomatous central visual field defects. J Glaucoma. 2021;30(6):e285–91. doi: 10.1097/IJG.0000000000001844 33813563 PMC8169576

[18] Behera G, Nath A, Ramasamy A, Kaliaperumal S. Comparing static perimetry protocols of central field testing among patients with glaucoma. Optom Vis Sci. 2023;100(6):406–11. doi: 10.1097/OPX.0000000000002020 37129640

[19] Nishijima E, Fukai K, Sano K, Noro T, Ogawa S, Okude S, et al. Comparative analysis of 24-2C, 24-2, and 10-2 visual field tests for detecting mild-stage glaucoma with central visual field defects. Am J Ophthalmol. 2024;268:275–84. doi: 10.1016/j.ajo.2024.07.024 39094994

[20] Nakanishi H, Akagi T, Suda K, Hasegawa T, Yamada H, Yokota S, et al. Clustering of combined 24-2 and 10-2 visual field grids and their relationship with circumpapillary retinal nerve fiber layer thickness. Invest Ophthalmol Vis Sci. 2016;57(7):3203–10. doi: 10.1167/iovs.15-18798 27309624

[21] Mine I, Shoji T, Kumagai T, Yoshikawa Y, Kosaka A, Shinoda K. Central visual field sensitivity with and without background light given to the nontested fellow eye in glaucoma patients. J Glaucoma. 2021;30(6):537–44. doi: 10.1097/IJG.0000000000001764 33350657

[22] Kumagai T, Shoji T, Yoshikawa Y, Mine I, Kanno J, Ishii H, et al. Comparison of central visual sensitivity between monocular and binocular testing in advanced glaucoma patients using imo perimetry. Br J Ophthalmol. 2020;104(11):1258–534. doi: 10.1136/bjophthalmol-2019-315251 32152139 PMC7587224

[23] Wakayama A, Matsumoto C, Ayato Y, Shimomura Y. Comparison of monocular sensitivities measured with and without occlusion using the head-mounted perimeter imo. PLoS One. 2019;14(1):e0210691. doi: 10.1371/journal.pone.0210691 30653560 PMC6336334

[24] Wakayama A, Nomoto H, Chiba Y, Matsumoto C, Kusaka S. Effect of sensitivity disparity between the two eyes on pointwise monocular sensitivity under binocular viewing in patients with glaucoma. J Glaucoma. 2021;30(1):37–43. doi: 10.1097/IJG.0000000000001675 33290308 PMC7774818

[25] Garway-Heath DF, Poinoosawmy D, Fitzke FW, Hitchings RA. Mapping the visual field to the optic disc in normal tension glaucoma eyes. Ophthalmology. 2000;107(10):1809–15. doi: 10.1016/s0161-6420(00)00284-0 11013178

[26] Ferreras A, Pablo LE, Garway-Heath DF, Fogagnolo P, García-Feijoo J. Mapping standard automated perimetry to the peripapillary retinal nerve fiber layer in glaucoma. Invest Ophthalmol Vis Sci. 2008;49(7):3018–25. doi: 10.1167/iovs.08-1775 18378581

[27] Kanamori A, Naka M, Nagai-Kusuhara A, Yamada Y, Nakamura M, Negi A. Regional relationship between retinal nerve fiber layer thickness and corresponding visual field sensitivity in glaucomatous eyes. Arch Ophthalmol. 2008;126(11):1500–6. doi: 10.1001/archopht.126.11.1500 19001216

